# Optimisations and Challenges Involved in the Creation of Various Bioluminescent and Fluorescent Influenza A Virus Strains for *In Vitro* and *In Vivo* Applications

**DOI:** 10.1371/journal.pone.0133888

**Published:** 2015-08-04

**Authors:** Monique I. Spronken, Kirsty R. Short, Sander Herfst, Theo M. Bestebroer, Vincent P. Vaes, Barbara van der Hoeven, Abraham J. Koster, Gert-Jan Kremers, Dana P. Scott, Alexander P. Gultyaev, Erin M. Sorell, Miranda de Graaf, Montserrat Bárcena, Guus F. Rimmelzwaan, Ron A. Fouchier

**Affiliations:** 1 Department of Viroscience, Erasmus Medical Centre, Rotterdam, the Netherlands; 2 School of Biomedical Sciences, University of Queensland, Brisbane, Australia; 3 Department of Molecular Cell Biology, Section Electron Microscopy, Leiden University Medical Centre, Leiden, the Netherlands; 4 Erasmus Optical Imaging Centre, Department of Pathology, Erasmus University Medical Centre, Rotterdam, the Netherlands; 5 Rocky Mountain Veterinary Branch, Division of Intramural Research, National Institute of Allergy and Infectious Diseases, National Institutes of Health, Hamilton, MT, United States of America; 6 Leiden Institute of Advanced Computer Science, Leiden University, Leiden, the Netherlands; 7 Milken Institute School of Public Health, Department of Health Policy and Management, George Washington University, Washington, DC, United States of America; Johns Hopkins University—Bloomberg School of Public Health, UNITED STATES

## Abstract

Bioluminescent and fluorescent influenza A viruses offer new opportunities to study influenza virus replication, tropism and pathogenesis. To date, several influenza A reporter viruses have been described. These strategies typically focused on a single reporter gene (either bioluminescent or fluorescent) in a single virus backbone. However, whilst bioluminescence is suited to *in vivo* imaging, fluorescent viruses are more appropriate for microscopy. Therefore, the idea l reporter virus varies depending on the experiment in question, and it is important that any reporter virus strategy can be adapted accordingly. Herein, a strategy was developed to create five different reporter viruses in a single virus backbone. Specifically, enhanced green fluorescent protein (eGFP), far-red fluorescent protein (fRFP), near-infrared fluorescent protein (iRFP), *Gaussia* luciferase (gLUC) and firefly luciferase (fLUC) were inserted into the PA gene segment of A/PR/8/34 (H1N1). This study provides a comprehensive characterisation of the effects of different reporter genes on influenza virus replication and reporter activity. *In vivo* reporter gene expression, in lung tissues, was only detected for eGFP, fRFP and gLUC expressing viruses. *In vitro*, the eGFP-expressing virus displayed the best reporter stability and could be used for correlative light electron microscopy (CLEM). This strategy was then used to create eGFP-expressing viruses consisting entirely of pandemic H1N1, highly pathogenic avian influenza (HPAI) H5N1 and H7N9. The HPAI H5N1 eGFP-expressing virus infected mice and reporter gene expression was detected, in lung tissues, *in vivo*. Thus, this study provides new tools and insights for the creation of bioluminescent and fluorescent influenza A reporter viruses.

## Introduction

Influenza A viruses cause yearly epidemics in humans and are an important cause of worldwide morbidity and mortality [1]. Occasionally, avian influenza strains cross the species barrier and infect humans; avian H5N1 and H7N9 viruses continue to be responsible for human cases and deaths across the globe [2, 3]. Alternatively, novel influenza viruses can be introduced into the human population following a reassortment event between different virus subtypes, as was observed with the pandemic H1N1 (pH1N1) virus in 2009 [4]. In order to better understand the replication and pathogenesis of influenza viruses, numerous advanced molecular biology techniques have been developed. For example, the development of the influenza A virus reverse genetics system [5–7] provided a means to genetically alter the influenza A virus genome [8]. The relative ease at which the influenza virus genome can now be manipulated has facilitated the creation of bioluminescent and fluorescently labelled influenza virus strains. These ‘reporter viruses’ provide a unique opportunity to study the spread of influenza viruses in animal models in real-time, identify the specific cell types that are involved in virus replication and answer other fundamental research questions about influenza virus pathogenesis [8–11].

To date, both bioluminescent and fluorescent influenza A reporter viruses have been described [11–22]. The first generation of influenza reporter viruses were replication deficient. These systems were used extensively to study the packaging mechanism of influenza A virus [23–25], attachment patterns [26], the detection of neutralizing antibodies [27] and co-infections [28]. The first replication competent influenza A reporter virus was that of Manicassamy and colleagues [12], where GFP was introduced into the NS gene segment of influenza A/PR/8/34 (H1N1) (A/PR/8). This virus represented a significant advancement in influenza virus research, as it facilitated the microscopic analysis of infected tissue [10, 11, 29]. Fluorescent reporter viruses, in combination with microscopy, can be used to visualise the site of virus replication and morphological changes in the cell following infection. Indeed, Manicassamy and colleagues [12] successfully tracked the virus dynamics *in vivo* in mice. However, a clonal GFP positive virus was only obtained after plaque purification. Moreover, GFP was found to be unsuitable for live *in vivo* imaging, due to the low tissue penetration of the fluorescent signal and the high background. When the same strategy was employed to create an A/PR/8 strain expressing tRFP (TurboRFP), reporter expression was lost after two passages [13]. A/PR/8 NS_RFP (mCherry) virus was attenuated *in vitro* when compared to the wild-type virus [21]. Recently, Fukuyama *et*. *al*. [22] improved the pathogenicity and stability of NS reporter viruses to be more comparable to wild-type virus by serially passaging a NS_Venus virus in mice. Moreover, they produced Venus-expressing highly pathogenic avian influenza (HPAI) H5N1 virus. However, this virus contained the NS gene segment of A/PR/8 rather than the NS gene segment of a circulating H5N1 strain.

In order to address some of these limitations, others have generated bioluminescent influenza A reporter viruses. For example, *Gaussia* luciferase (gLUC) was introduced into the PB2 [16] and NA [17] gene segments of A/PR/8. Although both viruses showed attenuation *in vitro*, they were visualised in the lung during live *in vivo* imaging experiments. Alternatively, Tran and colleagues [18] inserted nano luciferase (nLUC) into the PA gene segment of influenza A/WSN/33 (H1N1). This virus showed very little attenuation *in vitro* and *in vivo*, was stable across five passages in MDCK cells and showed a clear signal in real-time *in vivo* imaging in mice [18]. Recently, this strategy was also used to produce a pH1N1_nLUC virus which was successfully used in ferrets for live *in vivo* imaging [19]. However, it is important to recognise that these bioluminescent viruses require a substrate to be administered in order to detect reporter activity, making the imaging procedure more laborious and increasing the cost of experiments [10]. Furthermore, the level of detail provided by these viruses is limited as they are unsuitable for microscopy, and therefore it is not possible to identify individual virus positive cells. A universal strategy developed for both fluorescent and luminescent reporter viruses provides an opportunity to rapidly generate the most appropriate reporter virus, across multiple subtypes to answer specific research questions.

Here, we provide a strategy which was used to create five different reporter viruses, using enhanced GFP (eGFP), far-red fluorescent protein (fRFP), near-infrared fluorescent protein (iRFP), gLUC and firefly luciferase (fLUC) in the same virus backbone. The levels of attenuation, reporter expression and stability of the reporter viruses were compared and this strategy was also used to generate pH1N1, HPAI H5N1 and H7N9 eGFP-expressing viruses. A selection of these viruses were then used for *in vivo* imaging experiments.

## Material and Methods

### 2.1 Construction of reporter viruses

Reporter virus constructs were cloned using the PA gene segment of influenza A viruses A/PR/8/34 (H1N1), A/Netherlands/602/09 (pH1N1), A/Indonesia/5/05 (H5N1) and A/Anhui/1/13 (H7N9). Expression plasmids for H7N9 PB2, PB1 and PA were cloned into the pCAGGS plasmid, kindly provided by Dr. A. Garcia-Sastre (Icahn School of Medicine, New York, U.S.A.).

Influenza A virus reporter constructs were assembled using the pCAGGS plasmid as a shuttle vector. First, the 5’ untranslated region (UTR) and PA gene of the respective influenza A viruses was inserted into pCAGGS. The 17-aa 2A proteolytic site from foot and mouth disease virus (FMDV) [30], kindly provided by Dr. D. Perez (University of Maryland, U.S.A.), was then cloned directly behind the PA coding sequence (CDS). The PA 3’ UTR was introduced by PCR. The UTR_PA_2A_UTR cassette was cloned into the modified pHW2000 vector as described previously [31]. PCR-based cloning was then used to introduce a Glycine-Serine-Glycine amino acid spacer (GSG) [32, 33] between the PA CDS and 2A. The desired reporter genes, eGFP (eGFP-N1, Clontech, Saint-Germain-en-Laye, France), fRFP (TurboFP635, Evrogen, Moscow, Russia), iRFP (iRFP713, plasmid no. 31857, Addgene, Cambridge, U.S.A), gLUC (Nanolight, Pinetop, U.S.A.) and fLUC (Promega, Leiden, the Netherlands), were introduced between 2A and the PA 3’ UTR.

The packaging and promoter regions of various reporter constructs were further modified. The length of the initial PA packaging region was based on data described by de Wit *et al*. [34] and contained the terminal 149 nt of the PA gene segment. This duplicated PA packaging region (dPR) was inserted between the reporter gene and the 3’ UTR. Constructs with a shorter version of this packaging region (sPR) [18, 23] were also generated. Furthermore, silent mutations were introduced into the original PA packaging region that was still present in the PA CDS (mPR). To this end, a sequence of 48 amino acids, with 46 possibilities to introduce silent mutations, was synthesized at BaseClear (Leiden, the Netherlands). Promoter-up mutations in the influenza A virus promoter region – 2UP and 3UP – were generated as described previously by Neumann *et al*. for plasmids pHL1102 and pHL1103, respectively [35].

To produce recombinant influenza A reporter viruses, 293T cells were transfected with 5 μg of each plasmid encoding the 7 gene segments of the A/PR/8, pH1N1, HPAI H5N1 or H7N9 virus and the PA reporter construct of interest using the calcium phosphate method as described previously [31]. Cells were washed with PBS once and refreshed with DMEM containing 2% FCS the day after transfection. If necessary, *Vibrio cholerae* neuraminidase (VCNA; 1 mU/ml) and L-1-Tosylamide-2-phenylethyl chloromethyl ketone (TPCK) treated trypsin (10 μg/μl) was added. When no virus was obtained after the first passage in MDCK cells, a second passage was performed. In order to efficiently rescue the eGFP-expressing H7N9 virus, pCAGGS expression plasmids containing PB2, PB1 and PA were co-transfected. 293T supernatants were harvested by low-speed centrifugation and used to inoculate a confluent layer of MDCK cells and/or embryonated chicken eggs as described previously [36]. The sequences of all constructs and viruses were confirmed using a 3130XL genetic analyser (Applied Biosystems, Bleiswijk, the Netherlands). All primer and plasmid sequences are available upon request.

### 2.2 Minigenome assay

Minigenome assays using fLUC as a reporter were performed as described previously for PA constructs that contained fluorescent reporters [37]. An eGFP based minigenome assay was used for PA constructs that contained luminescent reporters. The pSP72-PhuTmu construct described by de Wit et. al. [31] was used to clone eGFP-N1, flanked by the 5’ and 3’ UTR’s from the NS gene segment of A/PR/8, in an antisense orientation. Next, 293T cells were transfected with 1 μg of the PB2, PB1, PA and NP bidirectional constructs, together with 0.6 μg of the GFP minigenome plasmid. Approximately 16 hours later, cells were washed once with PBS and refreshed with DMEM containing 10% FCS. Cells were incubated for ~24 hours at 37°C and 5% CO_2_ and the percentage of eGFP positive cells was determined by Fluorescent Activated Cell Sorting (FACS) on a FACS Canto (BD Biosciences, Breda, the Netherlands).

### 2.3 Western Blot

MDCK cells were inoculated at a multiplicity of infection (MOI) of 0.1. Approximately 24 hours after inoculation, cells were washed once with PBS and lysed in hot lysis buffer (1% sodium dodecyl sulfate (SDS), 100 mM NaCl, 10 mM EDTA, 10 mM Tris-HCl pH 7.5). The samples were treated with 3x dissociation buffer (2% SDS, 0.01 dithiothreitol, 0.02 M Tris-HCl at pH 6.8) for 10 minutes at 96°C and loaded on a 12.5% SDS-polyacrylamide gel. Proteins were transferred to nitrocellulose membrane by electroblotting. Blots were blocked overnight at 4°C in PBS containing 5% non-fat dry milk. The next day blots were washed three times with PBS 0.05% Tween-20. To detect the PA protein, a monoclonal mouse antibody was used in a 1/40 dilution (kindly provided by Dr. A. Nieto, CNB, Spain). To detect M1 a monoclonal mouse antibody (IgG1, Clone Hb64; ATCC) was used in a 1/2000 dilution. Antibodies were diluted in PBS containing 5% non-fat dry milk. Blots were incubated for one hour at RT and washed three times with PBS 0.05% Tween-20. A secondary goat-anti-mouse HRP antibody (Dako, Heverlee, Belgium) was then used in a 1/2000 dilution, in PBS containing 5% non-fat dry milk, for one hour at RT. After washing three times with PBS 0.05% Tween-20 blots were developed using ECL WB detection reagent (GE Healthcare, Eindhoven, the Netherlands) and imaged with a Chemidoc MP imaging system (BioRad, Veenendaal, the Netherlands). Analysis was performed using Image Lab software (BioRad).

### 2.4 Reporter activity

Expression of eGFP was visualized using an Axiovert 25 IF microscope (Zeiss, Sliedrecht, the Netherlands) followed by quantitative analysis using FACS. For iRFP, infected cells were visualized using a LSM-700 confocal microscope with filter set 32 (Zeiss) and quantitative analysis was done by FACS using the APC-Cy7 channel. Expression of fRFP was visualized using fluorescent microscopy with filter set 00 (Zeiss) and by FACS using the PerCP-Cy5-5-A channel.

The reporter activity of the gLUC virus was determined using a *Gaussia* luciferase flash assay kit (Pierce, Etten-Leur, the Netherlands) according to the manufacturer’s instructions. The Luciferase assay system (Promega, Leiden, the Netherlands) was used for fLUC, according to the manufacturer’s instructions. Luminescence was measured on an Infinite F200 reader (Tecan, Giessen, the Netherlands) in flat 96-black well plates (Corning, Amsterdam, the Netherlands).

### 2.5 Virus titrations

The fifty percent tissue culture infectious dose (TCID_50_) in virus stocks was determined as described previously [31] using infection medium with a concentration of 1 **μ**g/**μ**l TPCK treated trypsin (Sigma). Infection medium consisted of EMEM (Lonza, Breda, the Netherlands) supplemented with 100 IU/ml penicillin (Lonza), 100 **μ**g/**μ**l streptomycin (Lonza), 2 mM glutamine (Lonza), 1.5 mg/ml sodiumbicarbonate (Cambrex, Wiesbaden, Germany), 10 mM Hepes (Cambrex) and non-essential amino acids (MP Biomedicals Europe, Illkirch, France).

### 2.6 Replication kinetics

Multistep replication kinetics were determined by inoculating a confluent monolayer of MDCK cells at a multiplicity of infection (MOI) of 0.001. One hour after inoculation, at time zero, cells were washed twice with PBS and fresh infection medium was added. At 0, 6, 12, 24, 48 and 72 hours after inoculation samples were taken and used to determine the virus titre, as described above.

### 2.7 Stability experiments

To test the *in vitro* stability of the different reporter viruses, three individually rescued viruses were blindly passaged ten times in MDCK cells. MDCK cells were inoculated with 20 μl of virus supernatant from the previous MDCK passage in 3 ml of infection medium. Virus from each passage was used to inoculate a confluent monolayer of MDCK cells, using a 1/10 dilution to determine the presence of the reporter. Approximately 24 hours after inoculation, cells were used for FACS (eGFP, fRFP and iRFP) or luciferase reporter assays (gLUC and fLUC) as described above.

### 2.8 Inoculation of mice

Mice were housed and experiments were conducted in strict compliance with EU directive 86/609/EEC on animal testing and the Dutch Experiments on Animal Act, 1997. Each cage contained 6 mice with sawdust as bedding. Enrichment was offered in the form of paper tissues. Food and water were checked daily and the facility was dark/light for 12 hours. The protocol was approved by an independent animal experimentation ethical review committee “Stichting DEC consult” (Erasmus MC permit number EUR3385). Stichting DEC considers the application and pays careful attention to the effects of the intervention on the animal, its discomfort, and weighs this against the social and scientific benefit to humans or animals. The researcher is required to keep the effects of the intervention to a minimum, based on the three R’s (Refinement, Replacement, Reduction). Animal welfare was monitored daily. All experiments with HPAI H5N1 viruses in mice were performed under DM-III conditions.

Female BALB/c mice (Charles River, Leiden, the Netherlands; 7–9 weeks old) were randomly distributed into the relevant experimenal groups. Mice were anaesthetized with 3% isoflurane (Pharmachemie B.V., Haarlem, the Netherlands) in O_2_ and inoculated intranasally with 50 μl of 10^5^ TCID_50_ of A/PR/8 reporter or wild-type viruses, 10^4^ TCID_50_ of the HPAI 2UP_PA_eGFP_dPR(H5N1) or HPAI H5N1 wild-type virus or PBS for the mock-infected group. Each group consisted of 24 mice with six mice for each time-point. Before inoculation with gLUC, iRFP or fRFP viruses, the imaging region of interest was shaved to prevent the fur from quenching the signal [38]. Mice were weighed daily; if the loss in bodyweight exceeded 20% mice were killed humanely via cervical dislocation. Reporter activity was determined at day 1, 3, 4 and 5 and virus titres were measured upon homogenising the organs of interest, as described previously [39].

### 2.9 Assessment of reporter activity *in vivo*

To visualise the reporter activity *in vivo*, mice were anaesthetized with 3% isoflurane in O_2_ and transferred to a custom made, transparent, airtight IVIS box with a black inlay (Stout, Rotterdam, the Netherlands). The box was filled with a maintenance concentration (3%) of isoflurane in O_2_ and transported to the IVIS Spectrum system (Perkin Elmer, Groningen, the Netherlands) for *in vivo* imaging. For mice inoculated with the gLUC virus, 300 μg of coelenterazine-SOL *in vivo* substrate (Nanolight, Pinetop, U.S.A.) was administered via the tail vein, prior to transfer to the IVIS box and subsequent imaging. Next, the mice were euthanized and the lungs were transferred to a 6-well equiglass cell culture plate (Molecular devices, Sunnyvale, U.S.A.) for imaging. Given the high turnover rate of the substrate, lungs from the gLUC virus-inoculated mice were injected with 100 μg of coelterazine-SOL substrate, via the trachea. Lungs were imaged using the IVIS spectrum; lungs from mice inoculated with fluorescent reporter viruses were additionally imaged using fluorescent microscopy.

### 2.10 *In vivo* stability

To test the *in vivo* stability of the reporter viruses MDCK cells were inoculated with lung homogenates at a MOI of 0.1. At approximately 24 hours after inoculation cells were washed twice with PBS-1% FCS. Cells were resuspended in cytofix-cytoperm (BD biosciences) and incubated for 20 minutes on ice. Subsequently they were incubated for 1 hour, on ice, with a monoclonal mouse antibody against NP (IgG2A, Clone Hb65; ATCC), diluted 1/50 in perm-wash buffer (BD Biosciences). Cells were washed once in perm-wash and incubated for 1 hour, on ice, with either polyclonal rabbit-anti-mouse FITC (Dako, Heverlee, Belgium) or goat-anti-mouse APC (BD Biosciences), diluted 1/100 in perm-wash. Cells were washed once with perm-wash, resuspended in PBS and FACS was performed. Data was analysed with FlowJo, version Vx.07 (TreeStar, Inc, Ashland, U.S.A.).

### 2.11 Immunohistochemistry

At the relevant time points after inoculation mice were euthanized and lungs were inflated with 10% formalin and embedded in paraffin. Lungs were subsequently sectioned and immunohistochemistry (IHC) NP-staining was performed as described previously [40]. To detect eGFP in the mice inoculated with HPAI H5N1 reporter virus, a GFP rabbit IgG polyclonal antibody (Life Technologies, Bleiswijk, the Netherlands) was used in a 1/100 dilution. Normal rabbit IgG (R&D systems, Abingdon, UK) was used in a 1/100 dilution as a control. Anti-Rabbit IgG HRP (Dako) was used in a 1/200 dilution as a secondary antibody.

### 2.12 Statistics

Statistical significance of virus titres was determined using the nonparametric Kruskal-Wallis test with a Dunns post-test where p<0.05 was considered significant. Statistical significance of bodyweight was determined using the two-way ANOVA with a Bonferroni post-test where p<0.05 was considered significant. All statistical analyses were performed using GraphPad Prism 5.00 for Windows (GraphPad Software, San Diego, CA, U.S.A.).

### 2.13 Live *in vitro* imaging

Confluent monolayers of MDCK cells, in a 5 cm poly-D lysine coated glass bottom culture dish (MatTek, Ashland, USA), were inoculated with the 2UP_PA_eGFP_sPR virus at a MOI of 1. The plate was transferred to a temperature controled 37–2 digital mini-incubator (Peecon, Germany) and imaged using the LSM-700 confocal microscope (Zeiss), using a 10x/0/45NA Plan-Apochromat objective. EGFP fluorescence was excited at 488nm and detected through a 500–550nm bandpass emission filter. Ten positions were selected and imaged every 30 minutes for 72 hours. At each position a 3x3 tilescan (3072x3072 pixels in total) was made, with a pixelsize of 625nm, resulting in an imaged area of 1.92x1.92nm. The sample was kept in focus through a reflection-based autofocus routine. The images were used to generate a movie using the Fiji ImageJ software package [41].

### 2.14 Correlative light and electron microscopy (CLEM)

MDCK cells were grown on sapphire discs that had been imprinted with a finder grid pattern according to the method described by McDonald et. al. [42]. At 24 hours after inoculation with 2UP_PA_eGFP_sPR virus at a MOI of 1 (or mock infection for the control samples), cells were fixed with 3% paraformaldehyde (PFA) in 0.1 M 60 mM piperazide-1,4-bis[2-ethanesulfonic acid], 25 mM HEPES, 2 mM MgCl2, 10 mM EGTA (PHEM) for 2 hours at room temperature. The fixative was subsequently replaced with 0.5% PFA-PHEM solution before imaging using an AF6000 microscope equipped with a 40x NA 1.3 oil immersion objective (Leica). By using an automated stage, the entire sapphire was imaged for eGFP using a YFP filter cube (Leica), as well as in bright field mode to visualize the grid pattern in order to enable an easier correlation after EM processing. Sapphires were then frozen with high-pressure freezing (HPF) using a Leica EM PACT2. The freeze substitution was performed using an AFS2 (Leica) with a freeze substitution processor robot following the protocol described in [43]. Samples were kept at -90°C in 0.1% uranyl acetate in acetone for 48 hours, after which the temperature was raised with 5°C/h to -45°C. The samples were subsequently washed three times with acetone and infiltrated for 16 hours with Lowicryl HM20 (Agar Scientific, Essex, UK) of increasing concentrations: 10%, 25%, 50%, and 75%. The temperature was then raised to -25°C (5°C/hour) and 100% Lowicryl was added, which was allowed to infiltrate for 10 hours. This was replaced twice by fresh 100% Lowicryl for 10 hours each. Polymerization was accomplished using UV-light at low temperatures, initially for 48 hours at -25°C, and then, after raising the temperature to 20°C (5°C/h), for an additional 48 hours. The carbon grid pattern on the blocks was then used to select a specific area and to locate this same area of the sapphire in the bright field image. Sections from this area (100 nm thick) were cut with a UC6 ultramicrotome (Leica) and were picked up on finder grids coated only with a carbon layer (Agar Scientific). The sections were also contrasted using uranyl acetate and lead-citrate. The carbon grid pattern on the blocks was then used to select a known area. Imaging was carried out in a Tecnai 12 BioTwin transmission electron microscope (FEI company, Eindhoven, the Netherlands) operating at 120kV and equipped with an Eagle 4k cooled slow-scan charge-couple device (CCD) camera (FEI Company). Overlapping images were taken at a magnification of 18500x, with a pixel size of 1.17 nm at the specimen level, to cover an area of approximately 54 by 113 μm and then joined together into a stitched image as described previously [44]. The cells in this mosaic image were then correlated with the fluorescent image using the cell shape as a reference. This allowed for an accurate matching of cells before and after EM preparation, showing which cells are infected in EM. EM images were analysed by an ultrastructural pathologist for signs of virus budding.

## Results

### 3.1 Optimization of influenza A virus PA reporter constructs

**Fig 1:**
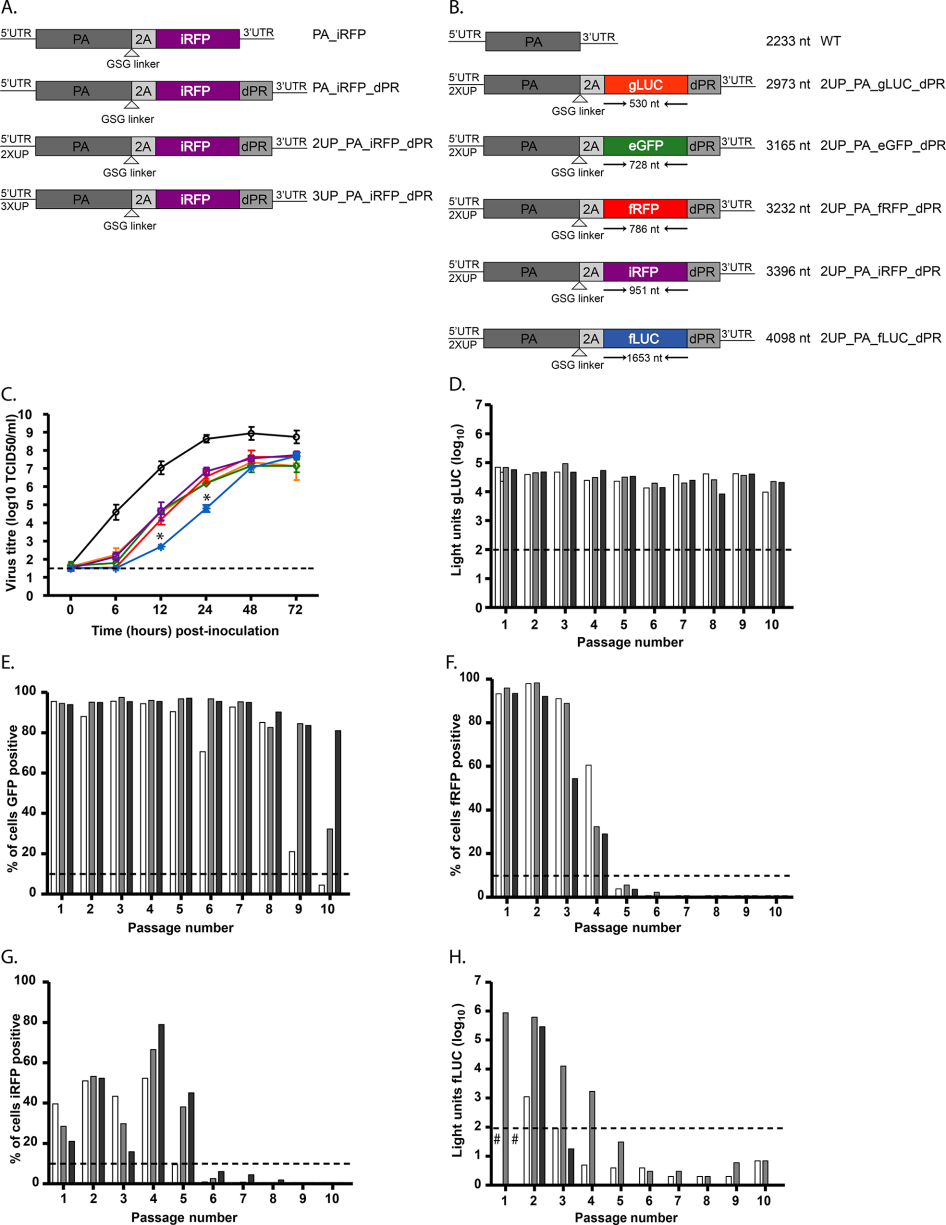
Optimisation and *in vitro* characterization of influenza A reporter viruses. (A) A schematic representation of the different cloning strategies used with iRFP as a representative fluorophore. All constructs contain a GSG spacer adjacent to the 2A cleavage site. Some constructs contain a duplicated packaging region (dPR) and promoter (2UP or 3UP) mutations. (B) A schematic figure of reporter viruses containing various fluorophores or bioluminescent proteins. The size of the reporter is indicated, as well as the total size of the construct and the construct name. (C) Replication kinetics of the reporter viruses as shown in panel B in MDCK cells at a MOI of 0.001 of WT (black circle), 2UP_PA_gLUC_dPR (yellow triangle), 2UP_PA_eGFP_dPR (green diamnd), 2UP_PA_fRFP_dPR (red cross), 2UP_PA_iRFP_dPR (purple square) and 2UP_PA_fLUC_dPR (blue star) virus. Error bars indicate the standard error of the mean. (D-H) Stability of reporter viruses: 2UP_PA_gLUC_dPR. (D), 2UP_PA_eGFP_dPR (E), 2UP_PA_fRFP_dPR (F), 2UP_PA_iRFP_dPR (G) and 2UP_PA_fLUC_dPR (H). A ^#^ indicates that no virus was produced in the first passage. For all reporter viruses 3 independently rescued viruses were selected and passaged 10 times in MDCK cells. The final passage was used to inoculate MDCK cells and after 24 hours the percentage of fluorescent positive cells or luciferase activity was determined. A threshold of ~3x the background values was applied and is indicated with a dotted line. Data were interpreted qualitatively.

To optimize the strategy to make an influenza A reporter virus, different constructs were cloned using iRFP as a representative fluorophore (Fig 1A). Firstly, a construct consisting of the PA 5’ UTR, the PA CDS without stop codon, a GSG spacer, the 17 aa proteolytic 2A sequence, iRFP and the 3’ UTR was produced. This construct was then further modified by inserting a duplication of the packaging region (dPR). Finally, mutations in the promoter region (2UP and 3UP) were introduced. A/PR/8 viruses were rescued with the reporter construct of choice and used to inoculate MDCK cells and embryonated chicken eggs. Virus titres from representative experiments and reporter activities from three individually rescued viruses are shown in Table 1.

**Table 1:** Optimisation of reporter constructs using iRFP as a representative fluorophore. Virus titres and reporter expression in MDCK cells or embryonated chicken eggs are shown. Virus titres were determined 3 days post-inoculation. For the reporter expression the mean and standard deviation is shown for 3 replicates based on individually rescued viruses after inoculation of MDCK cells at a MOI of 0.1.

Sample	TCID_50_/mlMDCK	% iRFP positive (MDCK)	TCID_50_/mlEgg	% iRFP positive (Egg)
**NC**	ND	0.7%±0.3	ND	0.7%±0.4
**PR/8 WT**	10^8.6^	0.7%±0.2	10^9.6^	0.6%±0.2
**PR/8 2UP_WT**	10^6.6^	0.8%±0.2	10^7.2^	0.8%±0.4
**PA_iRFP**	NR	ND	NR	ND
**PA_iRFP_dPR**	10^8.9^	0.07%±0.1	10^9.5^	0.6%±0.3
**2UP_PA_iRFP_dPR**	10^6.5^	13.3%±7.3	10^6.9^	0.9%±0.4
**3UP_PA_iRFP_dPR**	NR	ND	NR	ND

**NR**: not rescued, **ND**: not done, **NC**: negative control, **WT**: wild-type

Duplication of the packaging region (dPR) was essential to rescue iRFP reporter virus efficiently, whilst introduction of the 3UP mutation did not result in recombinant virus production. Although viruses that only contained the dPR had virus titres similar to the WT virus, no iRFP expression was detected. In contrast, the 2UP_PA_iRFP_dPR construct was the only one that resulted in recombinant virus expressing iRFP in MDCK cells, although the virus titres were lower than that of the wild-type. A reduction in virus titre was also observed when the 2UP mutation was introduced into the wild-type PA gene segment. Interestingly, none of the reporter viruses resulted in iRFP expression upon inoculation of MDCK cells when egg-grown viruses were used.

The 2UP_PA_iRFP_dPR cloning strategy was subsequently used to insert a selection of different fluorescent and luminescent reporters into A/PR/8, including gLUC, eGFP, fRFP and fLUC (Fig 1B).

### 3.2 Protein expression and recombinant virus production

The PA-reporter plasmids, as shown in Fig 1B, were used to assess if the presence of a reporter gene affected PA protein functionality. Minigenome assays were performed using eGFP minigenomes for luminescent PA and fLUC minigenomes for fluorescent PA. All PA-reporter constructs showed minigenome activity. The eGFP minigenome assay showed no difference between WT, WT_2UP and the luciferase PA constructs (S1A Fig). The fLUC minigenome assay was more variable and showed, in general, a lower minigenome activity when the eGFP PA construct was used (S1B Fig). Together this suggested that the different PA-reporter plasmids, most likely, produced enough PA protein for recombinant virus production.

Next, the different A/PR/8 reporter viruses were rescued and the virus titres of MDCK and egg-grown viruses were assessed in MDCK cells (Table 2). When MDCK cells were inoculated with the different reporter viruses, a large variation in virus titres was observed. The 2UP_PA_fLUC_dPR virus had the lowest virus titre, followed by the 2UP_PA_gLUC_dPR, 2UP_PA_iRFP_dPR, 2UP_PA_eGFP_dPR and 2UP_PA_fRFP_dPR viruses. No direct correlation between virus titre and length of the reporter gene was observed. All reporter viruses showed 0.5–4 log_10_ lower virus titres when compared to the wild-type virus. To confirm that the 2UP mutation was necessary for improved reporter expression (as was observed for the 2UP_PA_iRFP_dPR virus), reporter expression levels were compared to those of viruses without the 2UP mutation (S2A–S2D Fig). Without the 2UP mutation, only PA_gLUC_dPR and PA_eGFP_dPR virus showed substantial reporter expression. All viruses showed an increase in reporter expression level when the 2UP mutation was introduced. Consequently, the use of the 2UP mutation was beneficial for all reporters tested.

**Table 2:** Comparison of different PA reporter constructs. Virus titres and reporter activity in MDCK cells and embryonated chicken eggs are shown. For the reporter expression, the mean and standard deviation is shown for 3 replicates based on individually rescued viruses. Reporter activity is depicted by the percentage of expression or light units.

Sample	TCID_50_/mlMDCK	Reporter activityMDCK	TCID_50_/mlEgg	Reporteractivity egg
**NC**	ND	0.7%±0.3	ND	0.7%±0.4
**PR/8 WT**	10^8.6^	0.7%±0.2	10^9.6^	0.6%±0.2
**2UP_PA_gLUC_dPR**	10^6.2^	1.1x10^5^±8.0x10^3^	10^8.2^	6.6x10^4^±4.8x10^4^
**2UP_PA_eGFP_dPR**	10^6.7^	89.6%±1.9	10^8.1^	62.3%±40.2
**2UP_PA_fRFP_dPR**	10^8.0^	67.8%±6.3	10^7.1^	8.8%±15.2
**2UP_PA_iRFP_dPR**	10^6.5^	13.3%±7.3	10^6.9^	0.8%±0.3
**2UP_PA_fLUC_dPR**	10^4.7^	2.6x10^6^±3.1x10^6^	10^8.0^	7.8x10^5^±1.4x10^6^

**ND**: not done, **NC**: negative control, **WT**: wild-type

The reporter activity of the different viruses was further assessed using three independently rescued viruses produced in MDCK cells or embryonated chicken eggs (Table 2). The 2UP_PA_eGFP_dPR virus displayed the highest activity, whilst 2UP_PA_iRFP_dPR virus showed limited reporter expression. There were notable differences in luciferase expression of the independently rescued 2UP_PA_fLUC_dPR viruses. Interestingly, inoculation of MDCK cells with the egg-grown viruses resulted in no or very unstable reporter expression, although virus titres were comparable to the wild-type virus. Therefore, all subsequent experiments were performed using viruses amplified in MDCK cells.

To examine whether there was a correlation between the virus titre and the level of PA protein expression, MDCK cells were inoculated with the complete set of reporter viruses. After 24 hours, the levels of PA and M1 protein were compared using western blot (S1C Fig). The 2UP_PA_fLUC_dPR virus showed a large reduction in PA protein expression whilst the other viruses produced levels comparable to the wild-type virus. When PA protein expression was compared to the expression of M1, to normalise for the amount of virus present, the 2UP_PA_fLUC_dPR virus had the lowest and 2UP_PA_fRFP_dPR and wild-type viruses the highest ratio. These ratios were then compared to the virus titres to assess a possible association between virus titre and PA expression. The WT and 2UP_PA_fRFP_dPR virus had a high virus titre of ≥10^8.0^ and showed a PA-M1 ratio of above 1. The 2UP_PA_gLUC_dPR, 2UP_PA_eGFP_dPR and PA_iRFP_dPR viruses had intermediate virus titres of 10^6.2^–10^6.7^ and showed a PA-M1 ratio of 0.81–0.95. The 2UP_PA_fLUC_dPR virus had a low virus titre of 10^4.7^ and showed a PA-M1 ratio of 0.64 (Table 2 and S1C Fig). Thus, there was an association between the PA-M1 protein ratio and the titres of the respective reporter viruses. The reduced virus titres of the reporter viruses may thus result from a lower expression of PA protein. Nevertheless, these data demonstrate that the strategy to produce influenza A reporter virus that was developed using iRFP, can be successfully applied to other reporters.

### 3.3 *In vitro* characterisation of influenza A reporter viruses

Having established that the reporter viruses still produced functionally active PA, we then sought to determine if the reporter viruses were attenuated *in vitro* when compared to the wild-type virus. Accordingly, the replication kinetics of the reporter viruses were determined in MDCK cells (Fig 1C). At all time-points after inoculation (6, 12, 24, 48 and 72 hours) the reporter viruses displayed a trend towards a lower virus titre than the wild-type virus. The 2UP_PA_fLUC_dPR virus displayed a statistically significant (p<0.05) lower virus replication at 12 and 24 hours post-inoculation. However, at 72 hours post-inoculation the largest difference in virus titre between the wild-type and reporter viruses was 1.6 log_10_. Thus, although the reporter viruses were attenuated in a multi-step replication curve, they still reached an average peak virus titre of 10^7.5^ log_10_.

To assess the stability of each reporter virus, three individually rescued viruses were serially passaged ten times in MDCK cells and the fluorescent or bioluminescent activity was determined (Fig 1D and 1H). The 2UP_PA_gLUC_dPR and 2UP_PA_eGFP_dPR viruses were the most stable as 2UP_PA_gLUC_dPR was stable up to passage ten for all viruses and two out of the three 2UP_PA_eGFP_dPR viruses were also stable up to passage ten. The 2UP_PA_fRFP_dPR and 2UP_PA_iRFP_dPR virus stably expressed the reporter up to passage four and five, respectively. All three 2UP_PA_iRFP_dPR viruses showed an increased percentage of iRFP positive cells at passage four. The 2UP_PA_fLUC_dPR virus showed the lowest stability as only one virus showed luciferase activity until passage four. Furthermore, the other two viruses already lost luciferase expression at passage three. Overall, a large difference in stability for the different reporter viruses was observed and only a weak correlation between the stability of the different reporter viruses and the relative size of the reporter gene was observed.

Previous studies have shown that the process of plaque purification improved reporter virus stability [12, 13]. The 2UP_PA_eGFP_dPR virus was used to test whether plaque purification enhanced stability. Three rounds of plaque purification were performed in duplicate. From each round three viruses were selected to passage ten times in MDCK cells, together with a non-plaque purified virus. Plaque purification did not contribute to the stability of the 2UP_PA_eGFP_dPR virus (data not shown); therefore all subsequent experiments were performed using non-plaque purified virus. Seven of the 19 viruses from the plaque purification experiment showed ≤ 35% of eGFP-expressing cells at passage 10 and the PA gene segment of these viruses was completely sequenced to investigate the cause of the loss in reporter expression (S3 Fig). Most viruses contained one or more mutations in the PA CDS and one virus had a mutation in eGFP. In six viruses, deletions were observed in the part of the PA gene segment that contained the reporter. One virus also contained an insertion of a small part of eGFP in the dPR region. However, these deletions were present at different locations and varied in size.

### 3.4 Optimisations of influenza A reporter viruses

**Fig 2:**
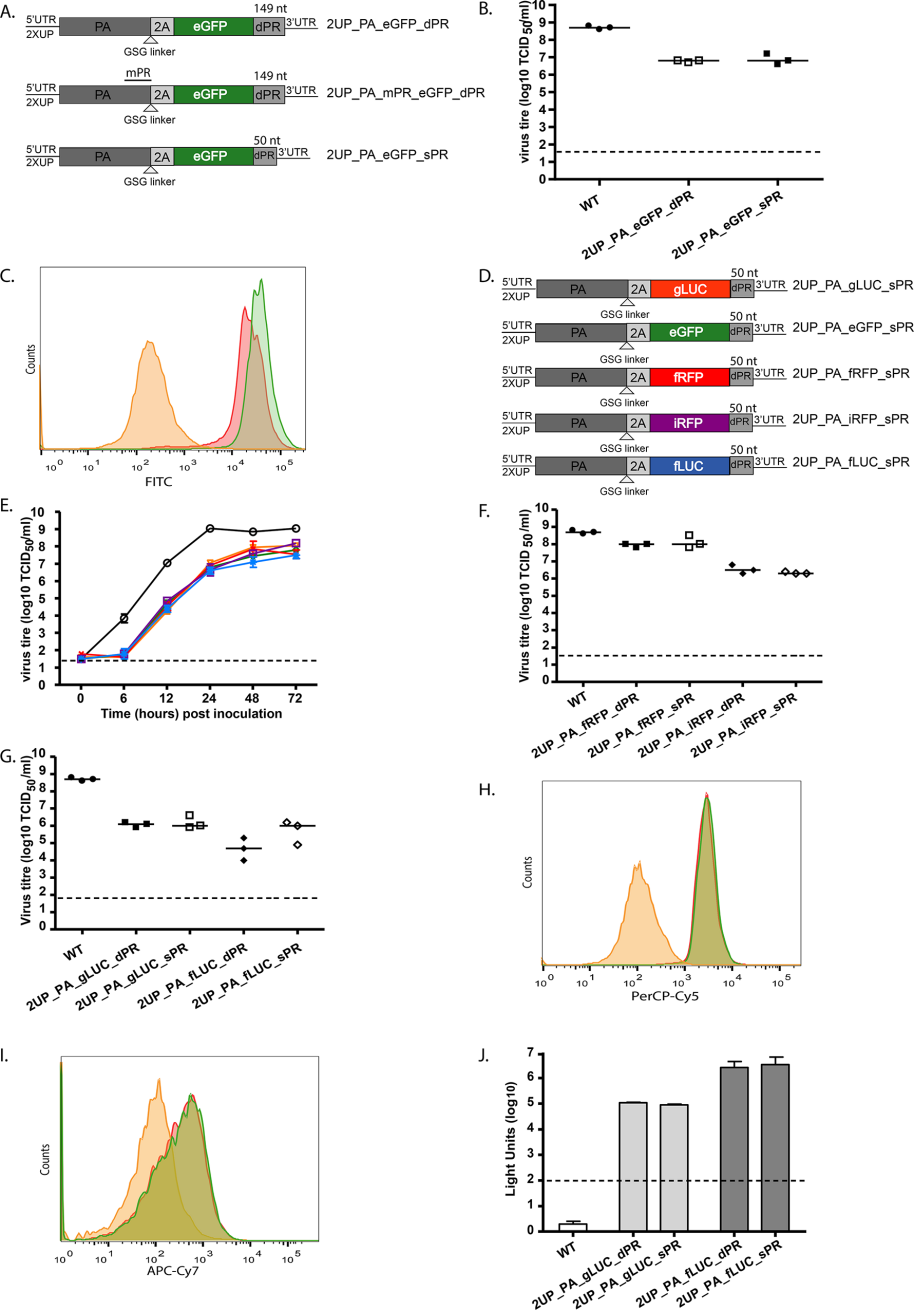
Further optimisation and *in vitro* characterization of influenza A reporter viruses. (A) A schematic representation of adjustments to the 2UP_PA_eGFP_dPR construct. Silent mutations were introduced into the PA packaging region present in the PA CDS (mPR) and the dPR was decreased from 149 to 50 nucleotides (sPR). (B) Virus titres of the wild-type and eGFP reporter viruses based on data from three individually rescued viruses. Data are represented by x-y scatter plots showing individual virus titres, the bars represent the median and the dotted line the detection limit. (C) eGFP expression levels were determined, the negative control (yellow square), 2UP_dPR (red square) and 2UP_sPR (green square) are indicated. Data are a representative of 3 independent experiments. (D) A schematic figure of reporter viruses containing various fluorophores or bioluminescent proteins with the sPR. (E) Replication kinetics of the sPR reporter viruses upon inoculation in MDCK cells at a MOI of 0.001 of WT (black circle), 2UP_PA_gLUC_sPR (yellow triangle), 2UP_PA_eGFP_sPR (green diamond), 2UP_PA_fRFP_sPR (red cross), 2UP_PA_iRFP_sPR (purple square) and 2UP_PA_ffLUC_sPR (blue star) virus. (F-G) Virus titres of the wild-type, fRFP and iRFP (F) reporter viruses and gLUC and fLUC (G) reporter virus based on data from three individually rescued viruses. Data are represented by x-y scatter plots showing individual virus titres, the bars represent the median and the dotted line the detection limit.(H-I) The expression levels for the fRFP (H) and iRFP (I) reporter virus were determined. The negative control (yellow square), 2UP_dPR (red square) and 2UP_sPR (green square) are indicated. Data are a representative of 3 independent experiments. (J) Intensity of reporter expression for the luminescent viruses based on data from three individually rescued viruses. Data represent mean expression levels and error bars indicate the standard error of the mean. The dotted line represents the threshold. Statistical significance was determined using the Kruskal-Wallis and Dunn’s multiple comparison test.

Recently, Tran et. al. [18] developed an A/WSN-based nLUC PA-reporter virus that displayed very little attenuation. This reporter virus differed from those created in this study. Firstly, silent mutations were introduced into the terminal part of the PA CDS to avoid an exact duplication of the packaging region in the reporter construct. Secondly, a smaller dPR consisting of 50 nt was used, rather than the 149 nt used in the present study. Given that the viruses used in this study were attenuated when compared to the wild-type virus, the 2UP_PA_eGFP_dPR construct was used for further optimisations. Firstly, silent mutations, as described by Tran et. al. [18], were introduced into the PA CDS to yield 2UP_PA_mPR_eGFP_dPR. Secondly, the dPR at the 3’ end was decreased in size from 149 nt to 50 nt (short packaging region; sPR) to yield 2UP_PA_eGFP_sPR. An overview of these constructs is shown in Fig 2A. The 2UP_PA_mPR_eGFP_dPR virus did not result in enhanced reporter expression or virus replication (data not shown). The 2UP_PA_eGFP_sPR virus showed a similar virus titre, but enhanced eGFP expression levels were observed (Fig 2B and 2C). Therefore, all PA-reporter constructs (both fluorescent and bioluminescent) with the sPR were cloned (Fig 2D).

The replication kinetics of the sPR reporter viruses were measured in MDCK cells to determine the attenuation levels *in vitro* (Fig 2E). The reporter viruses demonstrated a lower virus titre compared to the wild-type virus at all time-points post-inoculation. At 72 hours post-inoculation the largest difference in virus titre between the wild-type and reporter viruses was 1.5 log_10_. This was comparable to the previously described set of reporter viruses (Fig 1C).

### 3.5 Influence of the sPR on expression level, virus titre and stability

The possible influence of the sPR on expression levels of the reporter and virus titres were assessed. MDCK cells were inoculated with the initial set of reporter viruses (as shown in Fig 1B) or the viruses with the sPR (as shown in Fig 2D) to determine virus titres (Fig 2F and 2G) and reporter expression (Fig 2C, 2H, 2I and 2J). All reporter viruses showed a decreased virus titre compared to the wild-type virus. With the sPR no effect on virus titre was observed; only the 2UP_PA_fLUC_sPR virus showed a slightly higher virus titre.

**Fig 3:**
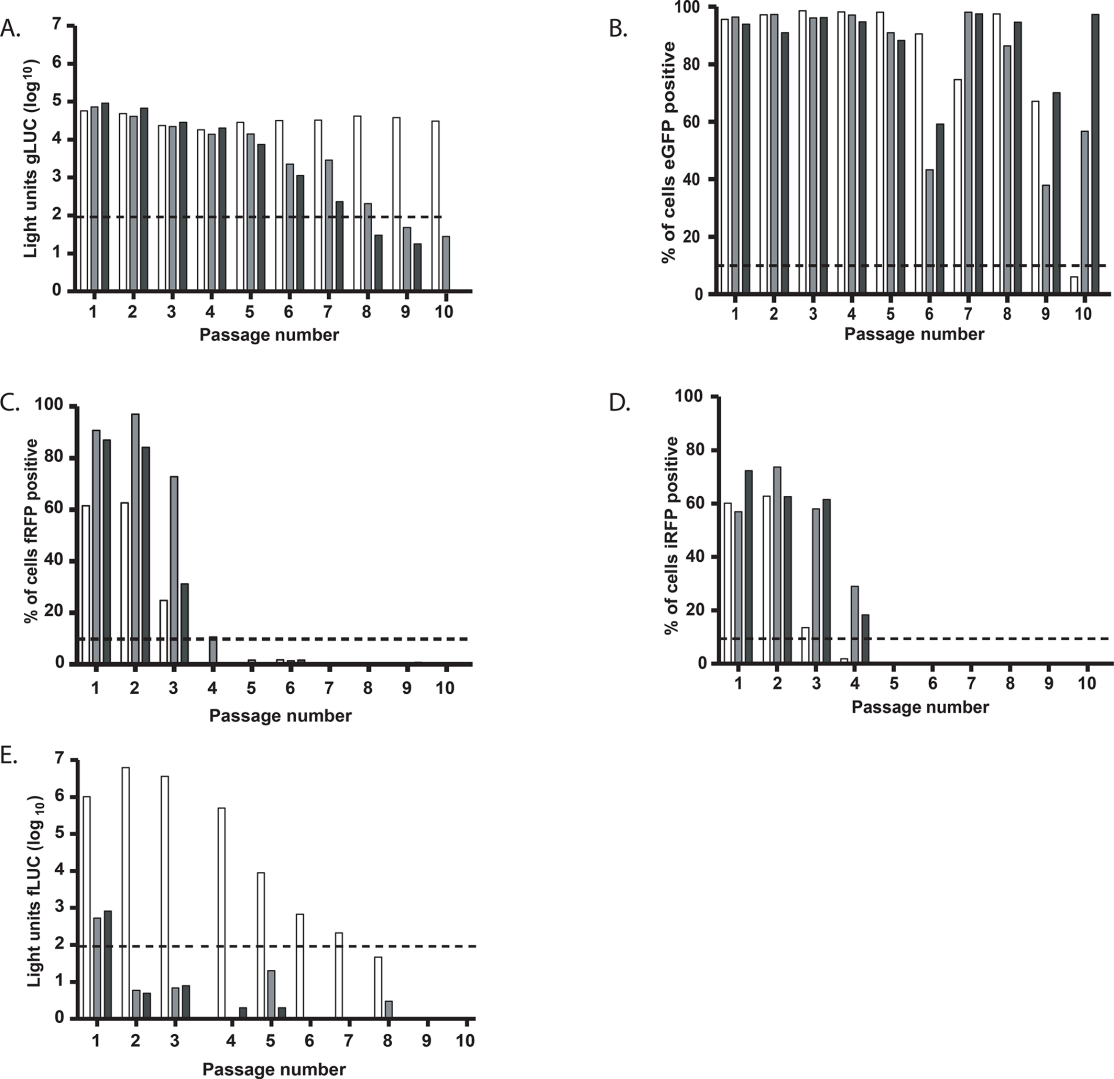
Reporter virus stability (A-E). Stability of reporter viruses containing the sPR for: 2UP_PA_gLUC_sPR (A), 2UP_PA_eGFP_sPR (B), 2UP_PA_fRFP_sPR (C), 2UP_PA_iRFP_sPR (D) and 2UP_PA_fLUC_sPR (E). For all reporter viruses 3 independently rescued viruses were selected and passaged 10 times in MDCK cells. The final supernatant was used to inoculate MDCK cells and after 24 hours the percentage of fluorescent positive cells or luciferase activity was determined. A threshold of ~3x the background values was applied and is indicated with a dotted line. Data were interpreted qualitatively.

The stability of the sPR reporter viruses was then assessed by passaging three individually rescued viruses ten times in MDCK cells (Fig 3A–3E). The 2UP_PA_gLUC_sPR (Fig 3A) and 2UP_PA_eGFP_sPR (Fig 3B) viruses were the most stable. However, the 2UP_PA_gLUC_sPR virus was slightly less stable compared to the 2UP_PA_gLUC_dPR virus (Fig 1D), as only one virus showed stable reporter expression up to passage ten. Reporter expression of the 2UP_PA_fRFP_sPR and 2UP_PA_iRFP_sPR was lost one passage earlier than the initial set of reporter viruses (Figs 1F, 3C, 1G and 3D). For one out of the three 2UP_PA_fLUC_sPR viruses (Fig 3E), stability was markedly increased as this virus remained stable up to passage seven (albeit with an apparent decrease in expression from passage four onwards). However, the other two 2UP_PA_fLUC_sPR viruses lost reporter expression at passage two. Overall, the 2UP_PA_gLUC_sPR, 2UP_PA_fRFP_sPR and 2UP_PA_iRFP_sPR viruses lost reporter expression approximately one passage earlier when compared to the original reporter viruses with the larger PR. Together, these data demonstrate that the sPR had a limited impact on virus replication and stability of the different reporter viruses.

As the sPR resulted in enhanced reporter expression for the eGFP virus but did not result in a decrease in reporter expression or have a major impact on stability for the other reporter viruses, sPR viruses were selected for *in vivo* experiments. Since the iRFP virus showed a very low percentage of cells that expressed the reporter, the passage four virus with the highest percentage of iRFP positive cells from the stability experiment (as shown in Fig 1G) was selected for *in vivo* experiments. The fLUC virus was not included in *in vivo* experiments due to the high level of attenuation.

### 3.6 *In vivo* experiments

**Fig 4:**
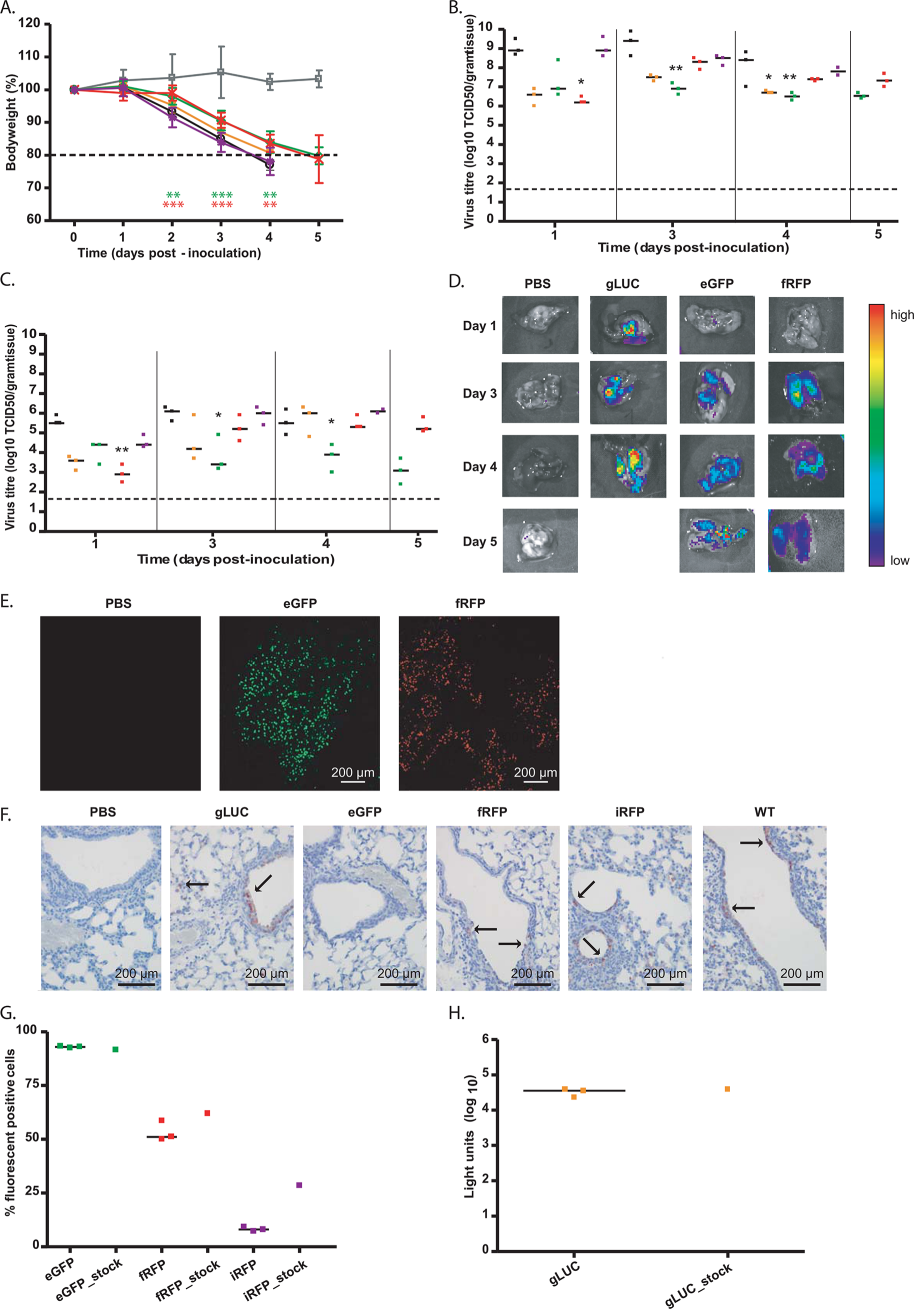
*In vivo* assessment of selected reporter viruses. (A) Loss of bodyweight after intranasal inoculation of mice with PBS (grey square), WT (black circle), 2UP_PA_gLUC_sPR (yellow triangle), 2UP_PA_eGFP_sPR (green diamond), 2UP_PA_fRFP_sPR (red cross) and 2UP_PA_iRFP_dPR (purple square). Statistical significance was determined using two-way ANOVA with Bonferroni post-test and is depicted by two (p<0.01) or three (p<0.001) asterisks. Error bars indicate the standard error of the mean. Each group consisted of 6 mice for each time-point. Colours of the asterisk represent the different reporters (green for eGFP and red for fRFP virus). Lung (B) and nose (C) samples from 3 mice were homogenized and used for virus titrations at day 1, 3, 4 and 5. Black squares represent wild-type, orange squares gLUC, green squars eGFP, red squars fRFP and purple squares the iRFP virus. Statistical significance was determined using the Kruskal-Wallis and Dunn’s multiple comparison test and is depicted by one (p<0.05) or two (p<0.01) asterisks. Data are represented by x-y scatter plots showing individual virus titres, the bars represent the median and the dotted line the detection limit. (D) Lungs were imaged using the IVIS Spectrum. Colours represent the intensity of the signal with purple indicating the lowest and red the highest signal. (E) Confocal images of the entire lungs from mice inoculated with PBS, 2UP_PA_eGFP_sPR and 2UP_PA_fRFP_sPR virus. (F) Sections of lungs were stained for NP antigen. The red colour represents NP positive cells and arrows indicate examples of positive cells. (G) Lung homogenates and virus stocks were used to inoculated MDCK cells to determine the *in vivo* stability. The cells were stained for NP and within the influenza A positive population the percentage of fluorescent cells or luciferase activity (H) from the respective reporter was determined. Data are represented by x-y scatter plots showing individual virus titres, the bars represent the median and the dotted line the detection limit.

To determine if the viruses produced in this study could be used *in vivo*, BALB/c mice were inoculated intranasally with 50 μl of 10^5^ TCID_50_ of wild-type A/PR/8 virus, 2UP_PA_gLUC_sPR, 2UP_PA_eGFP_sPR, 2UP_PA_fRFP_sPR or 2UP_PA_iRFP_dPR virus. The mock group was inoculated with 50 μl of PBS. Bodyweight was monitored daily and if the loss in bodyweight exceeded 20% the mice were killed humanely. Mice (*n = 6* per group) were imaged (*n = 3*) and virus titrations were performed (*n = 3*) at day 1, 3, 4 and 5 post-inoculation. All groups showed a reduction in bodyweight from day 2 onwards (Fig 4A). The reduction in bodyweight was statistically significant at day 2, 3 and 4 for the 2UP_PA_eGFP_sPR and 2UP_PA_fRFP_sPR group when compared to the mice inoculated with the wild-type virus. Mice inoculated with the 2UP_PA_gLUC_sPR, 2UP_PA_iRFP_dPR and wild-type virus showed ≥20% loss in bodyweight on day 4 whilst mice inoculated with the 2UP_PA_eGFP_sPR and 2UP_PA_fRFP_sPR virus reached this loss in bodyweight on day 5.

At all time-points, nose and lung tissues were homogenised and used for virus titrations. Virus was detected in the lung and nose from day 1 onwards for all reporter viruses and the wild-type virus (Fig 4B and 4C). At day 1, both the nose and lung tissues from mice inoculated with the 2UP_PA_fRFP_sPR virus had a statistically significant (p<0.01 and p<0.05 respectively) lower virus titre compared to the wild-type virus. At day 3 and 4, mice inoculated with the 2UP_PA_eGFP_sPR virus had significantly lower virus titres in both the nose and lung (p<0.05 and p<0.01 respectively) compared to mice inoculated with the wild-type virus. Mice inoculated with the 2UP_PA_gLUC_sPR virus also showed significantly (p<0.05) lower virus titres in the lung at day 4. At day 5, mice inoculated with the 2UP_PA_eGFP_sPR virus showed a very low virus titre in the nose but still high virus titres in the lung. Mice inoculated with the 2UP_PA_fRFP_sPR virus showed a similar virus titre as the eGFP reporter virus in the lung but a higher virus titre in the nose. Since the mice inoculated with the wild-type virus had to be euthanised at day 4, the virus titres obtained at day 5 could not be compared to the wild-type group. In general, mice inoculated with the 2UP_PA_iRFP_dPR virus most closely resembled replication of the wild-type virus.

To assess whether the different reporters could be detected *in vivo*, live imaging was performed using mice inoculated with the 2UP_PA_gLUC_sPR, 2UP_PA_fRFP_sPR and 2UP_PA_iRFP_dPR viruses, while under anaesthesia. The animals were imaged, together with a mock-infected animal, using the IVIS. Mice inoculated with the 2UP_PA_eGFP_sPR virus were not subjected to whole body imaging, as previous studies have shown that eGFP is unsuitable for *in vivo* imaging of the lung [12, 45]. Unfortunately, none of the reporter viruses showed a signal in real-time (data not shown). Next, the lungs were isolated and imaged using the IVIS (Fig 4D). At day 1, a positive signal was observed in mice inoculated with the 2UP_PA_gLUC_sPR virus. At day 3, mice inoculated with 2UP_PA_gLUC_sPR, 2UP_PA_eGFP_sPR and 2UP_PA_fRFP_sPR also showed a positive signal in the lung, which remained present until the end of the experiment. The strongest signal was observed with the 2UP_PA_gLUC_sPR virus, although this signal was not bright enough for whole body imaging. No signal was detected in the lungs of 2UP_PA_iRFP_dPR-inoculated mice. To determine whether this was caused by the detection limit of the IVIS or due to a loss of the iRFP gene, lung homogenates from day 1 and 4 were used to inoculate MDCK cells. This resulted in iRFP positive cells (data not shown), indicating that the lack of iRFP signal in the IVIS was caused by the low expression of iRFP rather than a loss of the reporter gene *in vivo*.

Next, the lungs from mice inoculated with fluorescent viruses were subjected to fluorescent microscopy. Fluorescent cells were present at day 1, 3, 4 and 5 with a strong increase between day 1 and 3. A representative picture, taken at day 3, for the lung area with the highest signal is shown in Fig 4E. Day 3 was selected since this represented the peak of replication for the majority of the viruses (Fig 4B and 4C). IHC for influenza virus antigen confirmed the presence of NP positive cells in the bronchiole and alveolus for all reporter viruses, except for 2UP_PA_eGFP_sPR virus (Fig 4F). However, the damaged appearance of the bronchiolar epithelium of these mice (data not shown) suggests the presence of recent viral infection, and that perhaps the time-point selected was too late to see a large number of NP positive cells. Nevertheless, these data demonstrate that the selected reporter viruses were able to replicate in mice.

To assess the *in vivo* stability of the different reporter viruses lung homogenates, from day 3, were used to inoculate MDCK cells at a MOI of 0.1. The corresponding MDCK virus stock was included as a control. The infected cells were stained for NP and a comparable percentage of infected cells was observed between MDCK cells inoculated with the lung samples and the virus stocks (data not shown). The NP-positive cell population was selected and the percentage of (fluorescent) reporter expression was determined (Fig 4G). The percentage of eGFP- and fRFP-infected cells was similar for all 3 lung samples and the virus stock. Only very limited iRFP expression was observed, both in the lung and MDCK stock samples. However, the percentage of iRFP-expressing cells was twice as high when the MDCK stock was used. For cells inoculated with the gLUC samples the luciferase activity was determined (Fig 4H), luciferase levels of the three lung samples and the virus stock were comparable. All reporter viruses that were detected in mice lungs using the IVIS showed *in vivo* stability of the reporter.

### 3.7 Applications

Influenza A reporter viruses are not only valuable for *in vivo* experiments but they can also be combined with a broad range of microscopy techniques [29, 46], as staining is not required to detect the presence of the virus.

The 2UP_PA_eGFP_sPR virus was thus used in a time-lapse fluorescent microscopy experiment. MDCK cells, cultured on a glass cover slip, were inoculated with the 2UP_PA_eGFP_sPR virus. Every 30 minutes, an image was obtained until 72 hours post-inoculation (S4 Fig). After ~6.5 hours the first cells became eGFP positive, with peak expression at ~19.5 hours. Cell death started to occur at ~30 hours.

**Fig 5:**
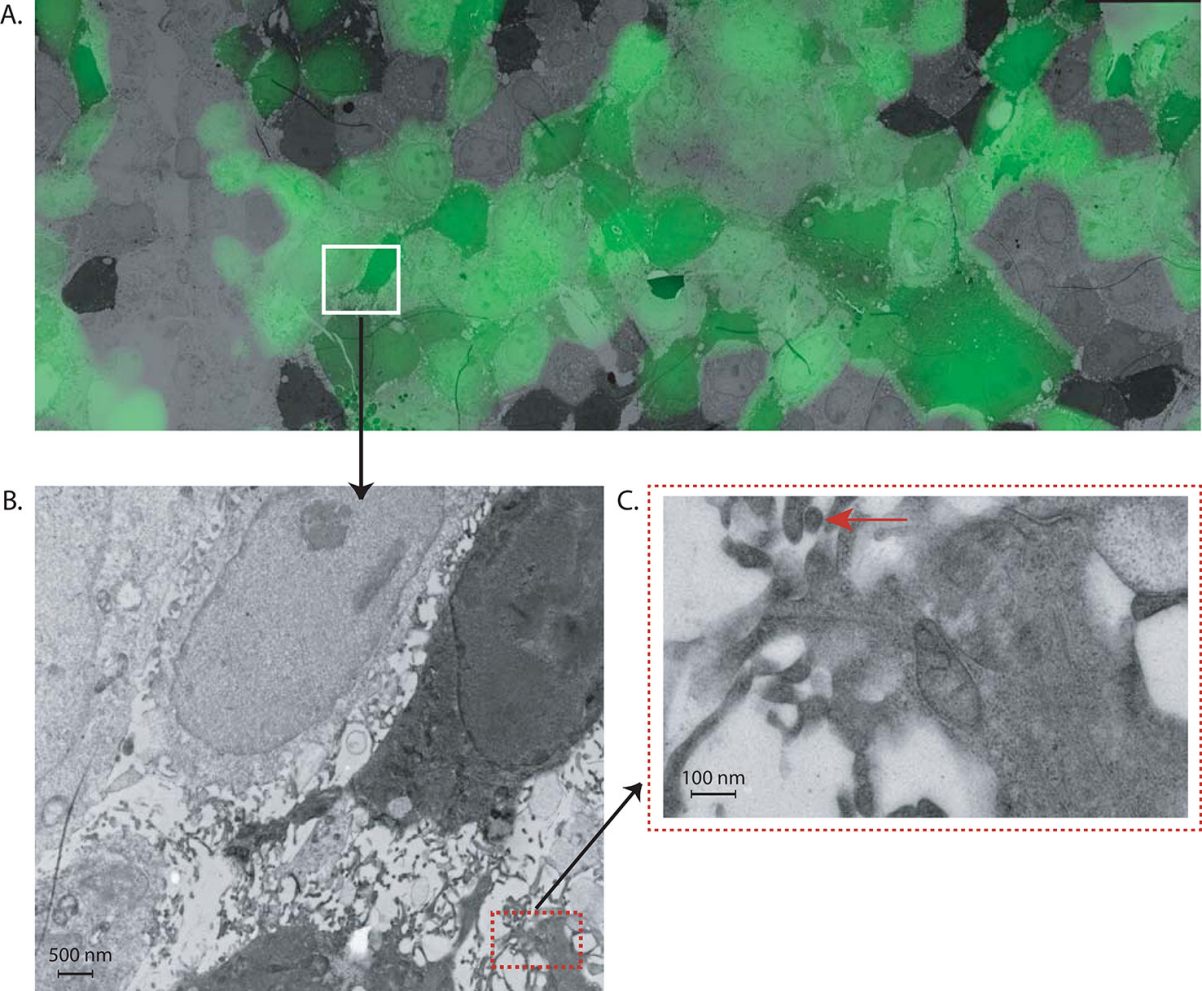
CLEM. Gridded sapphire discs with MDCK cells were inoculated with 2UP_PA_eGFP_sPR virus and processed for EM. (A) Overlay of the fluorescent signal with the EM stiched image. The white box contains an example of an area that was selected for further EM analyses based on the high level of eGFP expression (B) EM overview of a fragment of the selected area (white box of Fig 5A). (C) EM picture showing an example of a budding virus-like particle (indicated by the white arrow) present in the selected region.

The 2UP_PA_eGFP_sPR virus was also used to assess if influenza A reporter viruses could be used to detect morphological changes within infected cells, as identified by the presence of an eGFP signal. To this end, MDCK cells, cultured on gridded sapphire discs, were inoculated with the 2UP_PA_eGFP_sPR virus at a MOI of 1 or infection medium for the negative control. The carbon grid on the sapphire disc (S5 Fig) served to select an area that was subsequently used to combine the fluorescent and EM image of that location (Fig 5A). Fig 5B shows an example of a selected area (white box in Fig 5A) displaying a high eGFP signal, which also displayed putative virus-like particles as detected by EM. At multiple locations, arborizing microvillar projections were identified that were consistent with influenza virus budding. An example of a virus-like particle, budding from these microvillar projections, is depicted in Fig 5C (red arrow). The size of this particle was approximately 67 nm. This is consistent with the size of an influenza virus particle and these particles were not observed in the mock infected cells (data not shown). Together, these data showed that this reporter virus can successfully be used to follow virus replication in real-time and for in depth morphological analysis of virus infected cells.

### 3.8 Reporter viruses derived from human virus isolates

The majority of reporter viruses, to date, have been produced in an A/PR/8 or A/WSN virus backbone [12–14, 16–18], with only one publication describing the virus rescue and *in vivo* imaging of a pH1N1 reporter virus [18]. Thus far, no detailed *in vitro and in vivo* experiments using a reporter virus derived, entirely, from the HPAI H5N1 virus and H7N9 virus have been described.

**Fig 6:**
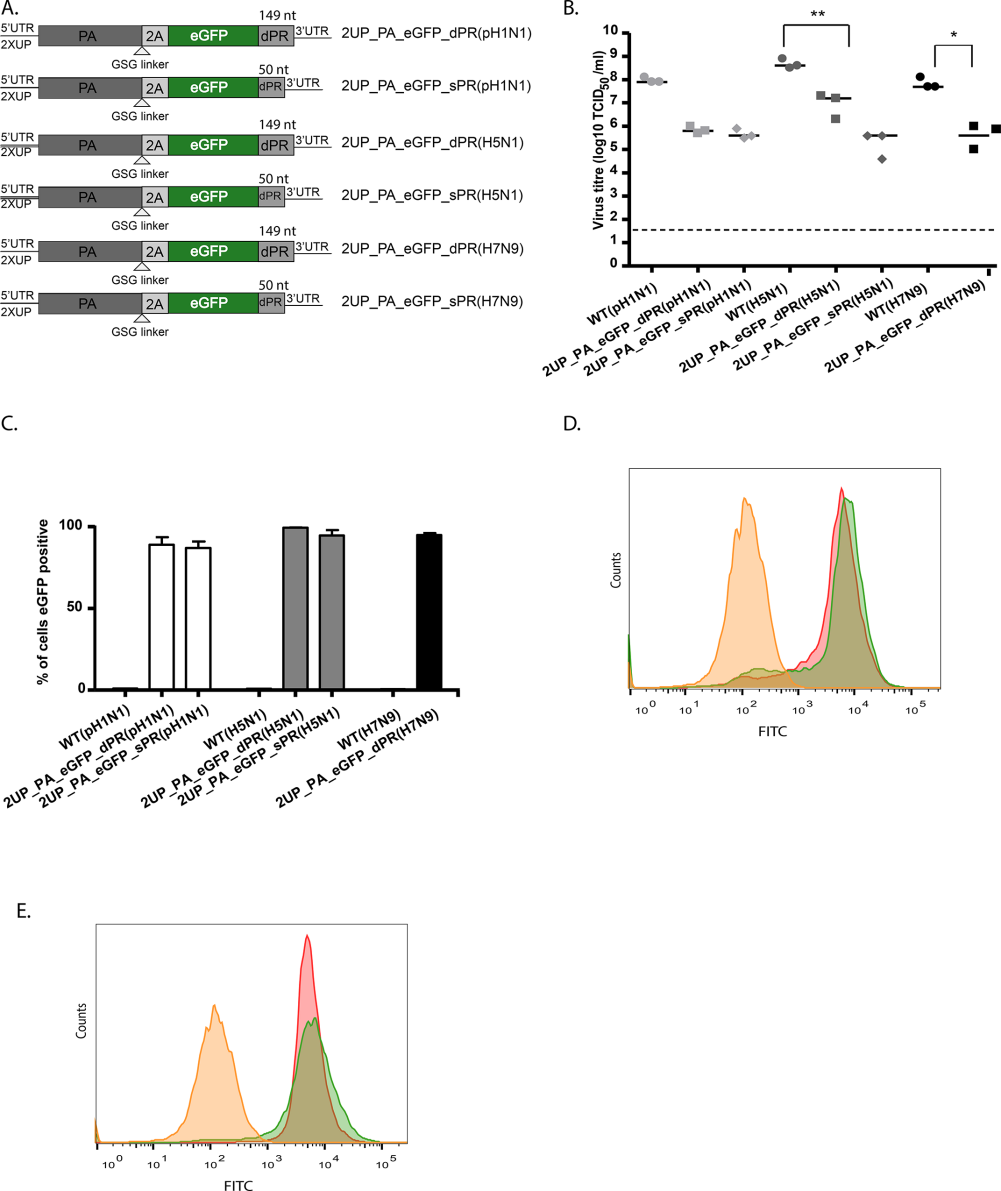
*In vitro* data of human eGFP-expressing viruses. Human pH1N1, HPAI H5N1 and H7N9 GFP reporter viruses were produced and compared to wild-type virus both *in vitro* and *in vivo*. (A) Schematic figure of eGFP reporter viruses from human influenza virus strains. (B) Virus titres are shown for wild-type and eGFP-expressing viruses with the 2UP mutation and, if rescued, the sPR virus. Statistical significance was determined using the Kruskal-Wallis and Dunn’s multiple comparison test and is depicted by one (p<0.05) or two (p<0.01) asterisks. Data are represented by x-y scatter plots showing individual virus titres, the bars represent the median and the dotted line the detection limit. (C) The expression level of the reporter was determined for the pH1N1, HPAI H5N1 and H7N9 eGFP viruses. Three individually rescued viruses were used to inoculate MDCK cells at a MOI of 0.1, after 24 hours eGFP expression was determined by FACS. Data represent mean expression levels and error bars indicate the standard error of the mean. (D) eGFP expression levels of pH1N1 and (E) H5N1 were determined. The negative control (yellow square), 2UP_dPR (red square) and 2UP_sPR (green square) are indicated. Data are a representative of 3 independent experiments.

The strategy described in this study was thus applied to a selection of viruses, consisting of pH1N1, HPAI H5N1 and H7N9 (Fig 6A) that expressed eGFP as the reporter of choice as this was the best performing, fluorescent, reporter. Wild-type PA, 2UP_PA_eGFP_dPR and 2UP_PA_eGFP_sPR of pH1N1, HPAI H5N1 and H7N9 were transfected with the remaining 7 matching gene segments to produce recombinant virus. All viruses, except for 2UP_PA_eGFP_sPR(H7N9), were rescued. Virus titres are shown in Fig 6B. For the 2UP_PA_eGFP_dPR(pH1N1) and 2UP_PA_eGFP_sPR(pH1N1) virus there was a reduction in virus titre of approximately 2 log_10_, compared to the wild-type pH1N1 virus. The HPAI 2UP_PA_eGFP_dPR(H5N1) virus was reduced by 1.5 log_10_ compared to wild-type HPAI H5N1 virus, and the HPAI 2UP_PA_eGFP_sPR(H5N1) virus had a significantly (p<0.01) lower virus titre of approximately 3.5 log_10_. The 2UP_PA_eGFP_dPR(H7N9) virus had a significantly (p<0.05) lower virus titre of approximately 2 log_10_ compared to the wild-type H7N9 virus. Strikingly, a difference between the various virus subtypes was observed. For the pH1N1 virus, the data resembled that of A/PR/8 (Fig 2B), where no difference in virus titre was observed between the 2UP_PA_eGFP_dPR and 2UP_PA_eGFP_sPR virus. In contrast, HPAI 2UP_PA_eGFP_sPR(H5N1) had a lower virus titre than the 2UP_PA_eGFP_dPR(H5N1) virus and the wild-type HPAI H5N1. Interestingly, the 2UP_PA_eGFP_sPR(H7N9) virus could not be rescued. The sPR thus seems to have a subtype-dependent effect on virus rescue and replication.

These viruses were also used to determine reporter expression. All eGFP viruses showed ~90–100% of eGFP positive cells upon inoculation of MDCK cells (Fig 6C). Expression levels of eGFP were only slightly higher for the viruses with the sPR (Fig 6D and 6E).

**Fig 7:**
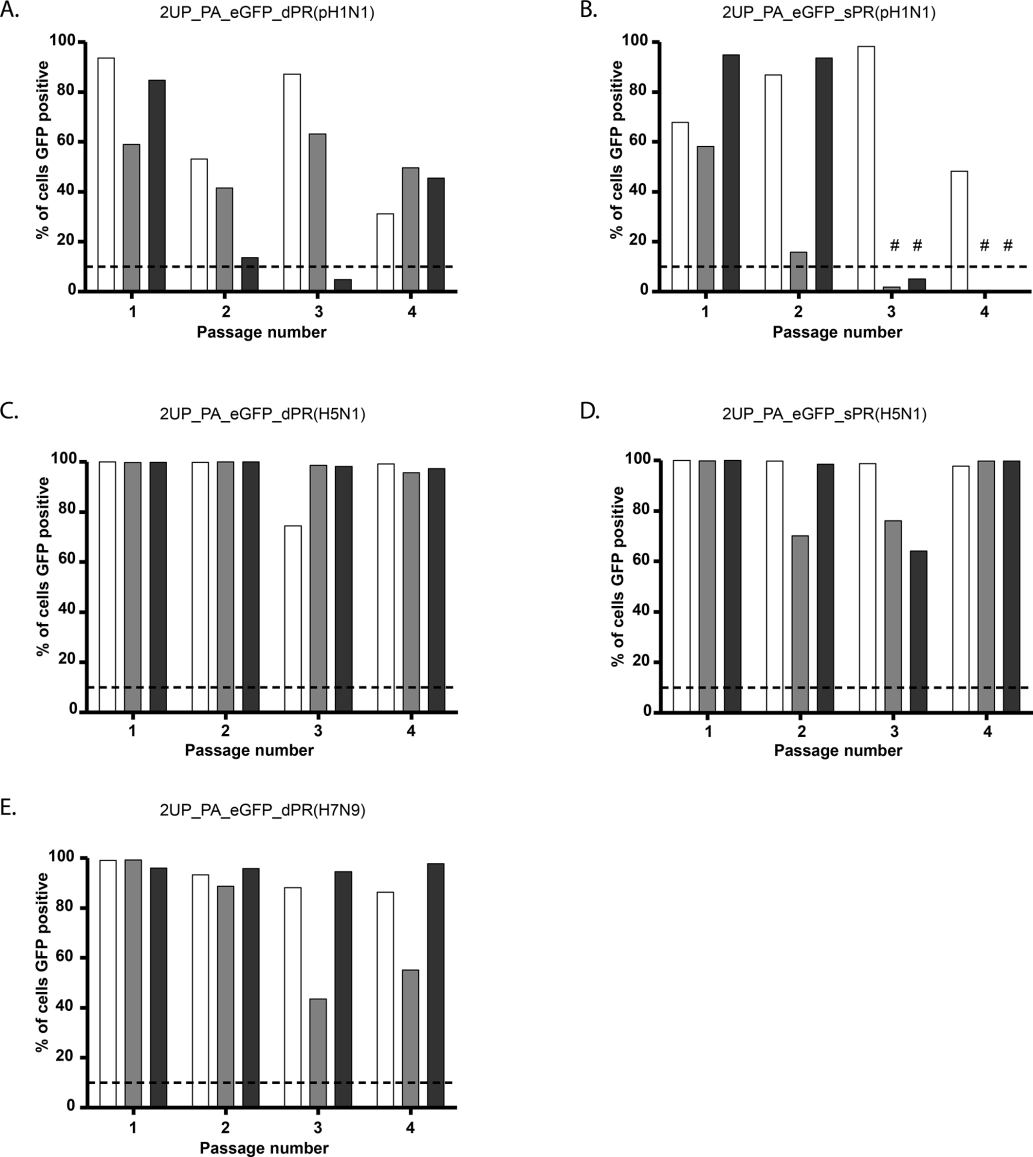
Reporter virus stability. **(A-E)** Stability of pHN1, HPAI H5N1 and H7N9 eGFP-expressing reporter viruses (A), 2UP_PA_eGFP_dPR(pH1N1) (B), 2UP_PA_eGFP_sPR(pH1N1) (C), 2UP_PA_eGFP_dPR(H5N1) (D) 2UP_PA_eGFP_sPR(H5N1) and (E) 2UP_PA_eGFP_dPR(H7N9). For all reporter viruses 3 independently rescued viruses were selected and passaged 4 times in MDCK cells. The final supernatant was used to inoculate MDCK cells and after 24 hours the percentage of eGFP-positive cells was determined. A threshold of ~3x the background values was applied and is indicated with a dotted line. Data were interpreted qualitatively. A ^#^ indicates that this sample was HA-negative.

Next, the *in vitro* stability of these reporter viruses was assessed. Three independently rescued viruses were selected for each virus and serially passaged four times in MDCK cells. For each passage, the percentage of eGFP-positive cells was determined (Fig 7A–7E). The 2UP_PA_eGFP_dPR(pH1N1) virus was stable up to passage four, although one virus showed very little or no eGFP-expression at passage two and three. From the 2UP_PA_eGFP_sPR(pH1N1) viruses only two out of three viruses replicated until passage two as no agglutination was observed in the HA assay at passage three and four. In general, the pH1N1 eGFP-expressing viruses were much less stable when compared to the A/PR/8 data (Figs 1E and 3B) and showed lower percentages of eGFP expression. Introduction of the sPR was detrimental to these viruses when passaged *in vitro*. The 2UP_PA_eGFP_dpR(H5N1), 2UP_PA_eGFP_sPR(H5N1) and 2UP_PA-eGFP_dPR(H7N9) viruses showed stable eGFP-expression up to passage 4. Together, these data demonstrate that the described strategy can be successfully applied to avian and human virus strains, although the pH1N1_GFP virus displayed limited *in vitro* stability.

### 3.9 *In vivo* experiments with HPAI H5N1 PA_eGFP

**Fig 8:**
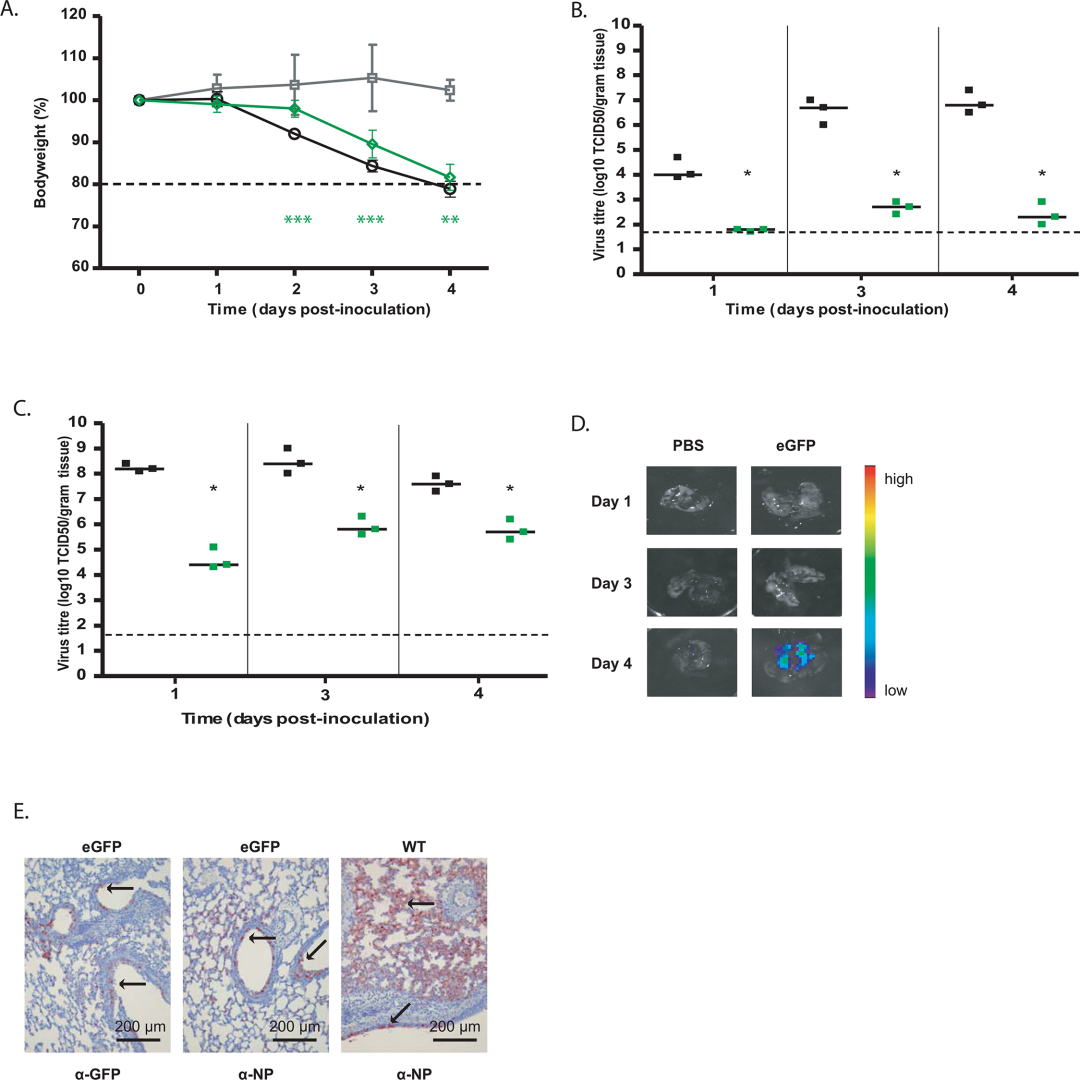
*In vivo* data of human eGFP-expressing viruses. (A) Loss of bodyweight after intranasal inoculation of mice with PBS (grey square), WT (black circle) and HPAI 2UP_PA_eGFP_dPR(H5N1) (green diamond) virus. Each group consisted of 6 mice for each time-point. Statistical significance was determined using two-way ANOVA with Bonferroni post-test and is depicted by two (p<0.01) or three (p<0.001) asterisks. Error bars indicate the standard error of the mean. The green colour represents the statistical significance of the HPAI 2UP_PA_eGFP_dPR(H5N1) virus. (B) Nose and (C) lung samples from 3 mice were homogenized and used for virus titrations at day 1, 3, 4 and 5. Black squares represent wild-type and green squares eGFP virus. Statistical significance was determined using the Kruskal-Wallis and Dunn’s multiple comparison test and is depicted by one (p<0.05) or two (p<0.01) asterisks. Data are represented by x-y scatter plots showing individual virus titres, the bars represent the median and the dotted line the detection limit. (D) Lungs were imaged using an IVIS spectrum. Colours represent the intensity of the signal with purple indicating the lowest and red the highest signal. (E) Sections of lungs were stained for NP and GFP antigen. The red colour represents NP or eGFP-positive cells and arrows indicate examples of positive cells.

To determine the *in vivo* characteristics of the HPAI 2UP_PA_eGFP_dPR(H5N1) reporter virus, BALB/c mice were inoculated intranasally with 50 μl of 10^4^ TCID_50_ of HPAI 2UP_PA_eGFP_dPR(H5N1), HPAI H5N1 wild-type virus or PBS. As the sPR construct had a negative influence on the virus titre, the HPAI 2UP_PA_eGFP_dPR(H5N1) virus was selected for *in vivo* experiments. Bodyweight was monitored daily and if the loss in bodyweight exceeded ≥20%, mice were killed humanely. Both groups lost bodyweight from day 2 onwards and at day 2, 3 and 4 a significant difference was observed for the HPAI 2UP_PA_eGFP_dPR(H5N1) virus when compared to the HPAI H5N1 wild-type virus (Fig 8A). At day 4, the mice from both groups had to be euthanised since they lost ≥20% of bodyweight.

Nose and lung tissues were homogenised and used for virus titration on day 1, 3 and 4 post-inoculation. The HPAI 2UP_PA_eGFP_dPR(H5N1) virus replicated to significantly (p<0.05) lower virus titres both in the nose and lung compared to the wild-type HPAI H5N1 virus (Fig 8B and 8C). Due to biosafety restrictions, the eGFP signal could only be visualised using the IVIS and IHC (Fig 8D and 8E). A clear eGFP signal was observed in lungs of HPAI 2UP_PA_eGFP_dPR(H5N1) infected mice at day 4 using the IVIS. Similarly, at day 3 post-inoculation, NP could clearly by visualised in the lungs of wild-type and HPAI 2UP_PA_eGFP_dPR(H5N1) infected mice by IHC (see Fig 8E). However, the NP antigen detected in HPAI 2UP_PA_eGFP_dPR(H5N1) mice was less diffuse and more focal in nature when compared to the wild-type HPAI H5N1. A similar pattern of staining was observed for the eGFP-antigen in the lungs of HPAI 2UP_PA_eGFP_dPR(H5N1)-inoculated mice, confirming the stability of the reporter gene *in vivo*. Together, these data demonstrate that whilst the HPAI 2UP_PA_eGFP_dPR(H5N1) virus is attenuated *in vitro* and *in vivo*, it still resulted in a detectable eGFP signal in mice and can thus be used for *in vivo* experiments.

## Discussion

Reporter viruses can be a valuable tool across a wide variety of research areas [10, 47, 48]. To date, several influenza A reporter viruses have been described [11–18, 21, 22, 49] but these strategies have yet to be tested back-to-back and across a broad range of reporter genes or virus backbones. Here, we provided an extensive, direct comparison of different reporter viruses both *in vitro* and *in vivo*. This strategy was then successfully applied to a set of relevant human virus isolates.

In this study, a 2UP mutation in the promoter region of the PA gene was necessary for successful reporter expression. Indeed, without this mutation only eGFP and gLUC were detectable in influenza A virus infected cells. A disadvantage of the 2UP mutation was that it resulted in lower virus titres (with the exception of the 2UP_PA_fRFP_dPR virus). When these mutations were introduced into the PA gene segment without a reporter, a similar decrease in virus titre was observed. This is consistent with the findings of Belicha-Villanueva and colleagues who showed that viruses with promoter mutations in the PB1 and PA gene segments of A/PR/8 displayed reduced replication *in vitro*, probably due to an increased production of non-infectious virus particles [50]. Interestingly, previous influenza virus reporter constructs produced reporter activity *in vitro* and *in vivo* in the absence of the 2UP mutation [12, 16–18]. This may suggest that the 2UP mutation only has an effect on the fluorescent proteins and bioluminescent markers tested in the present study. Nevertheless, in the reporter virus strategy described here it is imperative to use the 2UP promoter.

Another striking finding of the present study was that different reporters had a differential effect on virus attenuation and reporter activity. The selected reporters had a broad range in size with gLUC being the smallest (530 nt) and fLUC as the largest (1653 nt). Previous studies have shown that the coding capacity of the influenza virus gene segments is limited [51, 52]. Thus, it was expected that the size of the reporter gene would play a major role in replication efficiency and stability. Certainly, this appeared to be the case for the bioluminescent viruses. Viruses containing the relatively small gLUC reporter performed among the best both *in vitro* and *in vivo*. In contrast, viruses containing the relatively large fLUC had very low virus titres, were very unstable and showed a large variation in intensity if individually rescued viruses were compared. This is consistent with previous studies in alphavirus reporter systems [53]. However, this association between reporter size and virus attenuation/stability did not always hold true for the fluorescent viruses used in this study. For example, the fRFP (786 nt) reporter virus displayed equivalent stability to the iRFP (951 nt) reporter virus. Similarly, despite the presence of a much larger reporter gene, the 2UP_PA_iRFP_dPR virus had approximately the same virus titres as the 2UP_PA_eGFP_dPR virus. The reasons for these reporter gene dependent differences remain unclear. One possible explanation is that the inserted reporter has an effect on RNA structures present in the terminal part of the PA-reporter gene segment. The introduction of the reporter may result in RNA structures that are less stable and therefore affect virus replication. Indeed, preliminary data suggest that the folding free energy (ΔG) values of the predicted RNA structures at the termini of the different PA reporter constructs correlated with virus titres (data not shown). This remains an important area for future studies.

Interestingly, when compared to the recent publications by Tran *et*. *al*. [18] and Karlsson *et al*. [19] the PA-reporter viruses described here display increased attenuation and reduced utility for live *in vivo* imaging. This is in spite of the fact that the strategy described here was very similar to that of Tran and colleagues. This discrepancy may be due to study-dependent differences in the virus backbone, the characteristics of the selected reporter or interactions between the two. Indeed, this is consistent with the findings of the present study whereby the use of different reporters in the same virus backbone resulted in unpredictable stability and virus replication.

Unfortunately, a signal was not obtained during live *in vivo* imaging for any of the reporter viruses tested and all reporter viruses showed some level of *in vivo* attenuation. It remains possible that additional passaging of these viruses in mice would result in increased pathogenicity/reporter gene expression, as has recently been described by Fukuyama *et*. *al*. [22]. Alternatively, further optimisations to increase the signal of the reporter (potentially via the introduction of enhancer signals) [54, 55] may be necessary to facilitate live *in vivo* imaging. Such optimisations may also ensure that a lower viral doses could be used to infect mice and the pathogenesis of mild influenza virus infections could be examined. This remains an important area for future studies.

Despite these limitations, the fluorescent viruses created here could successfully be used to rapidly identify virus-infected cells *in vitro*. This then affords the researcher the opportunity to analyse, in depth, the morphology of infected cells and the virus particles which they produce using techniques such as CLEM. Moreover, the detection limit of microscopy is such that even areas with very few infected cells can be identified and then further processed using EM. This may be important to detect the early stages of virus infection and identify the actual site of replication to determine the associated cellular changes.

This study also provided a cloning strategy that could be used to create eGFP-expressing reporter viruses derived entirely from pH1N1, HPAI H5N1 and H7N9. To date, the majority of studies have focused on the creation of reporter viruses in laboratory strains of influenza (such as A/WSN/33 and A/PR/8), although recently a bioluminescent pH1N1 virus [19] and a Venus-expressing HPAI H5N1 virus [22] have been created. However, the Venus-expressing HPAI H5N1 virus contained the NS gene segment of A/PR/8, rather than that of the wild-type H5N1 virus. The generation of additional reporter viruses for human and avian influenza strains remains an important area of study, especially in light of the on-going introduction of new influenza virus strains into the human population.

Interestingly, there were striking virus dependent differences in the influence of the length of the packaging region on virus titre, eGFP-expression and stability in the various reporter viruses. For the pH1N1 eGFP virus the length of the PR had no effect on virus titre, as was seen for A/PR/8 eGFP virus, although they performed poorly in terms of *in vitro* stability. The percentage of eGFP-expressing cells was low and the sPR viruses lost the ability to replicate at passage three for two out of three viruses. However, for HPAI H5N1 eGFP a lower virus titre was observed with the decreased PR and for H7N9 eGFP no virus was rescued with the decreased PR. Both HPAI H5N1 and H7N9 e-GFP expressing viruses stably expressed eGFP until passage four. Previously, the minimal regions for optimal packaging were obtained using A/WSN and A/PR/8 viruses [23]. Here, the length of the packaging region was equal for all virus strains used. It is possible that the minimal packaging regions for HPAI H5N1 and H7N9 viruses are larger than for viruses of the H1N1 subtype. This would be surprising since the PA gene segment is highly conserved. RNA structures, most likely involved in packaging, may thus differ between subtypes. However, it is important to recognise that preliminary RNA structure predictions revealed no evidence for a decreased stability in HPAI H5N1 and H7N9 RNA structures when the PR was decreased (data not shown). This indicates that predicted RNA structures involved in packaging were not considerably distorted by the introduction of the eGFP reporter.

From the experiments described in this paper it is clear that a there is no “one size fits all” strategy to produce influenza A reporter viruses. Attenuation levels and stability are unpredictable and have to be assessed thoroughly before proceeding to *in vivo* experiments. However, the strategy developed here can clearly be used across a broad range of different reporter genes and virus backbones. This study thus provides a basis to continue to optimise influenza reporter viruses that can then be used to answer key questions in influenza A virus pathogenesis.

## Supporting Information

S1 Fig Minigenome and western blot. The PA reporter constructs were tested in a minigenome assay, using eGFP (A) or fLUC (B) minigenomes. (A). For the fLUC minigenome relative light units (fLUC divided by *Renilla*) were calculated. Error bars indicate the standard error of the mean based on data from three replicates. The white and black bars represent two independent experiments. MDCK cells were inoculated at a MOI of 0.1 and ~24 hours post-inoculation cells were harvested and loaded on a 12.5% SDS-PAGE gel. Antibodies against PA and M1 were used to detect the levels of protein produced (C). Numbers represent the ratio between the level of PA and M1 protein expression.
(EPS)

S2 Fig The 2UP mutation and expression level. The influence of the 2UP mutation on the level of reporter expression was determined for eGFP (A), fRFP (B) and iRFP (C) expressing viruses. For each virus a negative control (yellow square), noUP (blue square) and 2UP (red square) was compared. Data are a representative of 3 independent experiments. This was also done for the gLUC and fLUC expressing viruses. (D) Three individually rescued viruses were used to determine the expression levels for the luminescent viruses. Data represent mean expression levels and error bars indicate standard error of the mean. A threshold of ~3x the background values was applied and is indicated with a dotted line.
(EPS)

S3 Fig Sequence analysis of 2UP_PA_eGFP_dPR virus. A schematic representation of the 2UP_PA_eGFP_dPR gene segment showing the sequence analysis data from seven passage 10 MDCK-grown viruses. V1-V7 represent the different viruses that were sequenced. Vertical bars in the figure represent the nucleotide position of mutations or insertions (indicated by a red triangle). An asterisk shows if the mutation caused an amino acid change. Deletions are shown by horizontal bars and indicate the virus number, location of the deletion and the length of the deletion, which is shown in brackets.
(EPS)

S4 Fig Movie live *in vitro* imaging. A confluent monolayer of MDCK cells was inoculated with the 2UP_PA_eGFP_sPR virus at a MOI of 1. Time (hours post-inoculation) is depicted in the upper right corner.
(MOV)

S5 Fig Sapphire disc. Overview of a bright-field and eGFP image from the gridded sapphire disc. The dotted area represents the section that was used for further analysis by EM.
(EPS)
